# Food scarcity and decrease in income are associated with depression after COVID-19 pandemic in rural settings

**DOI:** 10.3389/fpubh.2025.1526300

**Published:** 2025-04-02

**Authors:** Dharmendra Gahwai, Sonal Dayama, Aakanksha Mishra, Sandip Kumar Chandraker, Babita Sahu, Mini Sharma, Ravindra Kumar

**Affiliations:** ^1^Directorate of Health Services, Raipur, India; ^2^ESIC Medical College, Hyderabad, India; ^3^Model Rural Health Research Unit, Durg, India; ^4^Pt. Jawahar Lal Nehru Memorial Medical College, Raipur, India; ^5^ICMR-National Institute of Reseach in Tribal Health, Jabalpur, India

**Keywords:** mental health, anxiety, depression, post-traumatic stress disease, COVID-19, rural area

## Abstract

**Objective:**

The current study is a cross-sectional survey that aims to assess an association COVID-19 on mental health in rural areas of Central India.

**Methods:**

Generalized Anxiety Disorder Assessment (GAD-7), Patient Health Questionnaire (PHQ-9), and Impact of Events Scale-Revised (IES-R) were used to evaluate the anxiety, depression, and post-traumatic stress disorder (PTSD) among families with at least one member having been affected by COVID-19 during November 2022 to December 2022 in Durg District of Chhattisgarh State.

**Results:**

A total of 431 participants were interviewed from 18 villages of Durg district of Chhattisgarh state. Symptoms of distress, anxiety and depression were observed in 26.2, 14.8 and 11.8% of participants. The death of family members due to COVID-19 and out of pocket expenditure was considerably associated with a higher risk of mental distress. A reduction in income was significantly associated with depression (*p*-value = 0.025, OR = 2.066, 95% CI = 1.115–3.817). Decline in income was also linked to depression among study participants (*p* value = 0.025, OR = 2.066, 95% CI = 1.115–3.817). Education, smoking and out of pocket expenditure was found be independently associated with occurrence of symptoms concerned with PTSD.

**Conclusion:**

The study points to the significance of socioeconomic factors like food security, and income stability during COVID-19 in mental health outcomes even after 1 year of pandemic. Increasing access to mental health resources and support for those affected by financial and food insecurities can help individuals cope with stress and maintain mental well-being.

## Introduction

Individuals affected by COVID-19 have experienced a range of mental health problems, including depression, anxiety disorders, panic attacks, irrational anger, impulsivity, sleep disorders, emotional disturbance, post-traumatic stress disorder (PTSD), and suicidal tendencies (1, 2). The global prevalence of anxiety and depression was increased by 25% in the first year of the COVID-19 pandemic (3). Further, during initial phase of the pandemic high prevalence of symptoms of anxiety (6.33–50.9%), depression (14.6–48.3%), post-traumatic stress disorder (7–53.8%) and psychological distress (34.43–38%) were reported in the general population during the COVID-19 pandemic across the world (4). While studies were indeed conducted during the onset of pandemic, to understand the immediate impact on mental health among various populations, it is crucial to continue studying the long-term effects and recovery, particularly in underrepresented areas such as rural communities. The mental health impacts of a crisis like a pandemic can extend far beyond the immediate crisis period.

Chhattisgarh, a state in India, has one of the highest suicide rates, with 28.2% compared to the national average of 12.4% (5). The state was also among the most affected by the COVID-19 pandemic, ranking twelfth in India (6). While numerous studies have been conducted on specific populations in India, very few have focused on the general population of rural settings. Therefore, a cross-sectional study was conducted in rural areas to determine the prevalence of depression, anxiety, and PTSD like symptoms among those affected with COVID-19 in the past 3 years and to identify the associated risk factors.

## Materials and methods

A community-based survey was conducted among rural and urban blocks of Durg district, Chhattisgarh, from November 2022 to December 2022. The demographic details of COVID-19 affected individuals were sought from the district hospital. Individuals who were affected from COVID-19 or individuals whose close family member was affected by COVID-19 or died due to COVID-19 in period between October to December 2021 were recruited from 18 villages. These 18 villages were selected conveniently from five blocks of the district for the study. For selecting participants, farthest house from the village was selected and then household were interviewed sequentially. Data was gathered from households that fulfilled the eligibility criteria.

### Sample size estimation

With a confidence level of 95%, assuming 50% prevalence from studies cited above, the sample size estimated was 384. Considering the non-response rate of 10%, the sample size estimated was 422. To achieve the sample size, 25 individuals were approached from each village.

### Data collection

Written informed consent was taken from each participant before the interview. The survey instrument consisted of two schedules. The first schedule was on sociodemographic data of the participant and his/ her family and medical history of the one affected with COVID-19, relevant personal history, hospital admissions or home isolation, presence of complications and health problems related to the disease etc. Those with preexisting mental illness (diagnosed before March 2020) were excluded from the study. The second schedule consisted of psychometric assessment scales, namely the Generalized Anxiety Disorder Assessment (GAD-7), Patient Health Questionnaire (PHQ)-9 and Impact of Event Scale-Revised (IES-R) scale. The GAD-7 schedule consists of seven items that are designed to identifying symptoms consistent with generalized anxiety disorder (GAD) (7). In order to complete the assessment, an individual is asked to rate the severity of their symptoms over the past 2 weeks. The PHQ-9 is the nine-item depression scale helpful in screening depression in primary care (8). It consists of nine questions that evaluate symptoms over the past 2 weeks. Each item is scored from 0 (not at all) to 3 (nearly every day), resulting in a total score ranging from 0 to 27. Higher scores indicate greater depression severity. Whereas IES-R is a self-report measure comprising 22 items; commonly used to symptoms related to subjective distress level related to traumatic event (9). The respondents are required to identify a specific stress-inducing life event and rank their level (scale 0–4) of distress or discomfort for each difficulty listed based on the past 7 days.

A cutoff score of >4 of PHQ9, >4 of GAD 7, and >23 for the IES-R scale was used to determine the presence of symptoms of depression, anxiety and PTSD, respectively.

### Statistical analysis

Data was presented as number and percentage for discrete values and mean ± SD for continuous variables. The Fisher exact test was applied to see the univariate association of different dependent variables with presence of symptoms of depression, anxiety and PTSD. Odds ratio (OR) with 95% confidence interval was also calculated to determine the strength of association. Multinomial logistic regression with backward elimination model was applied to see the independent risk factors for the symptoms of depression, anxiety and PTSD. A *p*-value of less than 0.05 was considered statistically significant. All the statistical analyses were conducted using IBM SPSS version 26 software for windows.

## Results

During the study, a total of 435 participants were recruited from 18 villages of Durg district and interviewed. Out of which, some information related to mental health was missing for four individuals and was excluded from the analysis. Therefore, analysis of the results was performed among 431 responses. The mean age of participants was 41 ± 14 years. There were 190 (44.1%) male and 241 (55.9%) female. Of these 431 participants, 157 (36.4%) have mental health issues (at least one of the three scales).

A total of 113 (26.2%, 43 males (22.6%) and 70 (29.0%) females) participants had symptoms concerned with PTSD. The mean IES-R scale score was 24.25 ± 6.98. Forty-four (10.2%) had moderate to severe symptoms (IES-R scores ≥33) (Supplementary Table 1). As expected, older peoples have higher occurrence of symptoms of PTSD than younger (Table 1). High prevalence of PTSD symptoms was observed in single participants as compared to married participants (*p* = 0.032, OR = 1.694, 95% CI = 1.055–2.719). Symptoms of PTSD was found in lesser number of participants in alcoholic and smokers’ groups as compared than non-alcoholic (*p* value = 0.028, OR = 0.559, 95% CI = 0.331–0.943) and non-smokers (*p* value = 0.009, OR = 0.460, 95% CI = 0.256–0.824) groups, respectively. The presence of comorbidity (*p* value = 0.048, OR = 1.551, 95% CI = 1.005–2.393) and death in the family due to COVID-19 (*p* < 0.001, OR = 4.893, 95% CI = 3.090–7.747) were associated with the symptoms of traumatic distress (Table 1). Out-of-pocket expenditure for medical expenses and shortage of food were also found to be associated with high PTSD in studied participants (Table 1). After applying multivariate logistic regression analysis Education, smoking and out of pocket expenditure was found be independently associated with occurrence of symptoms concerned with PTSD (Table 2).

**Table 1:** Association of mental health with socio-demographic factors.

Factors	Total	IES-R Score > 23 *n* (%)	OR (95% CI)	PHQ-9 Score > 4 *n* (%)	OR (95% CI)	GAD-7 Score > 4 *n* (%)	OR (95% CI)
Age group							
18–30 year	124	21 (16.9)	Ref	5 (9.8)	Ref	9 (7.3)	Ref
31–60 year	262	76 (29.0)	2.004 (1.168–3.439)^#^	36 (13.7)	3.791 (1.449–9.918)^#^	46 (17.6)	2.721 (1.286–5.758)^#^
> 60 year	45	16 (35.6)	2.706 (1.253–5.845)^#^	10 (22.2)	6.800 (2.179–21.220)^#^	9 (20.0)	3.194 (1.179–8.658)^#^
Gender							
Male	190	43 (22.6)	Ref	20 (10.5)	Ref	25 (13.2)	Ref
Female	241	70 (29.0)	1.399 (0.902–2.171)	31 (12.9)	1.255 (0.690–2.280)	39 (16.2)	1.274 (0.741–2.193)
Marital status							
Married	323	76 (23.5)	Ref	40 (12.4)	Ref	46 (14.2)	Ref
Single	108	37 (34.3)	1.694 (1.055–2.719)^#^	11 (10.2)	0.802 (0.396–1.626)	18 (16.7)	1.204 (0.664–2.183)
Education							
Up to primary	85	23 (19.8)	Ref	9 (7.8)	Ref	11 (9.5)	Ref
Middle to higher	230	58 (25.2)	0.909 (0.517–1.59)	29 (12.6)	1.218 (0.551–2.693)	34 (14.8)	1.167 (0.562–2.423)
Graduate	116	32 (37.6)	1.027 (0.548–1.925)	13 (15.3)	1.066 (0.433–2.622)	19 (22.4)	1.318 (0.591–2.939)
Employment/ Occupation							
Unemployed	117	22 (18.8)	Ref	9 (7.7)	Ref	16 (13.7)	Ref
Semi-skilled	176	57 (32.4)	2.068 (1.180–3.625)^#^	22 (12.5)	1.714 (0.759–3.868)	29 (16.5)	1.245 (0.643–2.414)
Skilled	138	34 (24.5)	1.414 (0.771–2.583)	20 (14.5)	2.034 (0.887–4.660)	19 (13.8)	1.008 (0.492–2.063)
Type of family							
Nuclear	262	62 (23.7)	Ref	24 (9.2)	Ref	37 (14.1)	Ref
Joint	169	51 (32.2)	1.394 (0.902–2.154)	27 (16.0)	1.886 (1.047–3.395)^#^	27 (16.0)	1.156 (0.675–1.982)
Family income							
Above Rs 20,000 per month	132	32 (24.2)	Ref	12 (9.1)	Ref	18 (13.6)	Ref
Rs. 10,001-Rs. 20,000 per month	153	34 (22.2)	0.893 (0.515–1.549)	19 (12.4)	1.418 (0.661–3.043)	20 (13.9)	0.952 (0.480–1.888)
Up to Rs. 10,000 per month	146	47 (32.2)	1.484 (0.874–2.516)	20 (13.7)	1.587 (0.743–3.388)	26 (17.8)	1.372 (0.714–2.638)
Public health insurance							
Yes	375	98 (26.1)	Ref	40 (10.7)	Ref	54 (14.4)	Ref
No	56	15 (26.8)	1.034 (0.548–1.951)	11 (19.6)	2.047 (0.980–4.276)	10 (17.9)	1.292 (0.615–2.715)
Place of residence							
Urban	158	44 (27.8)	Ref	19 (12.0)	Ref	23 (14.6)	Ref
Rural	273	69 (25.3)	0.876 (0.563–1.364)	32 (11.7)	0.971 (0.531–1.779)	41 (15.0)	1.037 (0.597–1.802)
Alcohol							
No	313	91 (29.1)	Ref	40 (12.8)	Ref	45 (14.3)	Ref
Yes	118	22 (18.6)	0.559 (0.331–0.943)^#^	11 (9.3)	0.702 (0.347–1.418)	19 (16.1)	1.143 (0.638–2.049)
Smoked tobacco							
No	331	97 (29.3)	Ref	44 (13.3)	Ref	49 (14.8)	Ref
Yes	100	16 (16.0)	0.460 (0.256–0.824)^#^	7 (7.0)	0.491 (0.214–1.127)	15 (15.0)	1.016 (0.542–1.901)
Chew tobacco							
No	345	90 (26.1)	Ref	39 (11.3)	Ref	48 (13.9)	Ref
Yes	86	23 (26.7)	1.034 (0.606–1.767)	12 (14.0)	1.272 (0.635–2.551)	16 (18.6)	1.414 (0.759–2.637)
Comorbidity							
No	259	59 (22.8)	Ref	34 (13.1)	Ref	33 (12.7)	Ref
Yes	172	54 (47.8)	1.551 (1.005–2.393)^#^	17 (9.9)	0.726 (0.392–1.345)	31 (18.0)	1.506 (0.883–2.564)
Casualty/Death in family due to COVID-19							
Yes	131	64 (49.2)	4.893 (3.090–7.747)^#^	17 (13.1)	1.167 (0.626–2.174)	25 (19.2)	1.578 (0.910–2.738)
No	300	49 (16.3)	Ref	34 (11.3)	Ref	39 (13.0)	Ref
Out of pocket expenditure*							
Nil	68	14 (20.6)	Ref	8 (11.8)	Ref	6 (8.8)	Ref
Up to Rs. 5,000	165	24 (14.5)	0.656 (0.316–1.363)	13 (7.9)	0.641 (0.253–1.626)	23 (13.9)	1.674 (0.649–4.315)
Rs. 5,000–10,000	39	10 (25.6)	1.330 (0.525–3.367)	4 (10.3)	0.857 (0.240–3.055)	4 (10.3)	1.181 (0.312–4.47)
Above Rs. 10,000	157	64 (40.8)	2.654 (1.360–5.180)^#^	26 (16.6)	1.489 (0.636–3.481)	31 (19.7)	2.542 (1.007–6.416)
Food scarcity during COVID-19							
Never	265	61 (23.0)	Ref	22 (8.3)	Ref	31 (11.7)	Ref
Sometime	105	28 (26.7)	1.216 (0.724–2.043)	20 (19.0)	2.599 (1.351–4.999)^#^	19 (18.1)	1.668 (0.895–3.108)
Often	61	24 (39.3)	2.169 (1.205–3.906)^#^	9 (14.8)	1.912 (0.832–4.391)	14 (23.0)	2.248 (1.111–4.549)^#^
Change in income							
No change	210	48 (22.9)	Ref	17 (8.1)	Ref	21 (10.0)	Ref
Decreased	221	65 (29.4)	1.406 (0.912–2.169)	34 (15.4)	2.066 (1.115–3.817)^#^	43 (19.5)	2.174 (1.241–3.808)^#^

* data of two participant was missing. # *p* value < 0.05.

**Table 2:** Multinomial logistic regression analysis.

**Variable**	***p* value**	**Adjusted OR**	**95% CI**
IES-R			
Education			
Graduate	0.020	1.964	1.112–3.468
Middle to higher	0.103	0.619	0.348–1.101
Upto primary	Ref	Ref	Ref
Smoking			
No	0.020	2.065	1.123–3.797
Yes	Ref	Ref	Ref
Out of pocket expenditure			
>10,000	0.003	2.782	1.402–5.520
5,000–10,000	0.457	1.434	0.555–3.705
upto 5,000	0.218	0.626	0.297–1.320
NIl			
PHQ9			
Age group			
>60 Years	0.000	12.587	3.599–44.029
31–60 Years	0.001	5.191	1.921–14.025
18–30 Years	Ref	Ref	Ref
Type of Family			
Joint	0.085	1.727	0.927–3.217
Nuclear	Ref	Ref	Ref
Public health insurance			
No	0.018	2.656	1.184–5.959
Yes	Ref	Ref	Ref
Smoking			
No	0.036	2.656	1.184–5.959
Yes	Ref	Ref	Ref
Co-morbidity			
Yes	0.057	0.506	0.251–1.021
No	Ref	Ref	Ref
Food scarcity during COVID-19			
Often	0.047	2.440	1.011–5.885
Sometime	0.001	3.411	1.668–6.977
None	Ref	Ref	Ref
GAD 7			
Age group			
>60 Years	0.014	3.539	1.290–9.712
31–60 Years	0.006	2.888	1.356–6.149
18–30 Years	Ref	Ref	Ref
Food scarcity during COVID-19			
Often	0.014	2.454	1.195–5.040
Sometime	0.096	1.706	0.909–3.203
None	Ref	Ref	Ref

The mean PHQ score was 1.32 ± 2.70. As per PHQ-9 score, total 51 (11.8%) participants were having symptoms of depression (Supplementary Table 1). Out of these, 45 participants had mild symptoms and four had moderate and one had severe depression. Older individuals were more likely of facing depression (Table 1). The occurrence of depression-like symptoms was higher in joint families compared to nuclear families (*p* value = 0.046, OR = 1.886, 95% CI = 1.047–3.395). Decrease in income was also found to be significantly associated with depression like symptoms (*p* value = 0.025, OR = 2.066, 95% CI = 1.115–3.817) (Table 1). Older age group, absence of public health insurance, smoking and scarcity of food during Covid-19 were the independent risk variables for the occurrence symptoms of depression in studied participants (Table 2).

A total 64 (14.8%) participants were found to have anxiety like symptoms as per GAD7 score. Fifty-three had mild anxiety symptoms (GAD7 score 5–9), seven had moderate symptoms (GAD7 score 10–14) and four had severe anxiety symptoms (GAD7 score > 15) (Supplementary Table 1). The mean score for GAD7 was 1.78 ± 3.04. The occurrence of anxiety symptoms was found higher in older age groups as compared to lower age group (Table 1). Participants who have shortage of foods during pandemic were more anxious (*p* value = 0.0371, OR = 2.248, 95% CI = 1.111–4.549). Further decrease in income was also found to be associated with anxiety symptoms (*p* value = 0.007, OR = 2.174, 95% CI = 1.241–3.808) (Table 1). Older age group and food scarcity was identified as independent risk factors for high GAD7 score (Table 2).

## Discussion

According to the current study, 26.2% of participants showed signs of PTSD, while 11.8% have depression, and 14.8% have anxiety symptoms after the COVID-19 pandemic. A systematic review and meta-analysis reported the pooled prevalence of anxiety 23.5% (95% CI: 17.4–29.6%) and 20.2% (95% CI: 17.2–23.2%) prevalence of depressive symptoms among general population of India during COVID-19 pandemic (10). Furthermore, a previous study conducted in urban Slum in North India observed the lower prevalence of depression (3.5, 95% CI = 0.95–6.05) and anxiety (2.5, 95% CI = 0.34–4.66) (11). Similarly, during the onset of the pandemic, there have been reports of relatively high rates of symptoms of anxiety, depression, and PTSD in various other countries (China, Denmark, Nepal, Spain, Italy, Iran, the US and Turkey) around the world during the COVID-19 pandemic. These symptoms have been reported with rates ranging from 6.33 to 50.9% for anxiety, 14.6 to 48.3% for depression, and 7 to 53.8% for post-traumatic stress disorder (2, 4, 12–16). Chronic disease patients, quarantined persons, and COVID-19 patients were at an increased risk of anxiety and depression than other populations (12).

Several risk factors associated with PTSD, depression and anxiety have been identified in various studies (17–24). Based on our study, it was found that the old age group was more likely to have symptoms of PTSD, depression and anxiety. In contrast, some studies have found the young population more prone to mental distress (13, 14), while reports on age as a risk factor were varying in other studies (17). Recently study conducted in central India showed the 7.83% prevalence of depression, 12.2% prevalence of anxiety and 5.2% prevalence of stress among older participants (25). In the current study, sex was not a significant risk factor. Other studies have found females to suffer more during the pandemic due to increased household responsibilities and domestic violence during the lockdown (18, 19, 22–24).

Food insecurity is a serious issue that can lead to significant stress and depression. Not knowing status of next meal creates a constant state of worry and anxiety, which can deeply affect mental health. In current study, food insecurity was found to be associated with depressed state. Previous studies have also shown that food insecurity is strongly associated with increased risks of depression, anxiety, and stress (26–28). For example, one study found that food insecurity was linked to a 257% higher risk of anxiety and a 253% higher risk of depression (28). However, all these studies were conducted in early days of pandemic. Public health strategies should focus on offering direct subsidies for food purchases to impoverished families and decreasing the stigma surrounding charitable food assistance.

The unpredictability and financial pressure can take a significant toll on mental well-being. Decrease in income was also found to be associated with depression and traumatic stress like symptoms in current study. Previous studies have also shown that individuals facing financial difficulties during the pandemic reported higher levels of psychosocial distress (29, 30). Another study reported that more than one-third of low-income adults screened positive for depression, anxiety, and high stress during the early days of the pandemic (27). Public health measures should focus on providing financial support and mental health resources to those affected by income loss. Previous studies also show that direct COVID-19 infection may not be the leading cause of long-term psychological distress. Instead, it appears that psychosocial factors related to the pandemic may be contributing to the increased prevalence of anxiety, depression and PTSD. Addressing the indirect effects of COVID-19 in long term (31) is crucial in mental health treatment and policy. The current study results provide important insights for mental health professionals and policymakers in developing strategies to prevent and treat PTSD.

Our study acknowledges some limitations, such as the self-reported information regarding co-morbidities. We did not analyze the association between the intensity or severity of mental health issues and the closeness or friendliness of the relative who succumbed to the pandemic. Additionally, we did not elicit the impact of severity of disease, such as hospitalization, ventilator usage, and near-death experiences, on mental status. We also did not capture social factors that may have influenced mental health status, such as income, financial or economic burden, food scarcity, and stigma, in detail through any standard tool. The effect of other variables such as social support, utilization of digital tools, access to healthcare which may impact the depression, anxiety and PTSD was not studied.

It is imperative to emphasize the significance of family focused therapy and community-based interventions such as providing mental health education to family and community members. Families play a crucial role in mental health treatment as supportive family relationships can enhance emotional well-being. Family-focused psychotherapeutic interventions can be applied to improve the mental health and well-being of individuals during these pandemic situations (32). Such measures are vital in creating a supportive environment, and we should continue striving to achieve these goals. To effectively address mental health during future pandemics, the healthcare system should incorporate mental health services into pandemic response plans, provide training to healthcare workers in psychological first aid, and establishing telehealth infrastructure (33) for remote mental health support. Family focused therapy, building social support networks and involving community leaders in promoting mental health awareness (34) can be vital steps in overcoming the challenges related to mental health. Furthermore, increasing mental health research and preparedness funding can help inform evidence-based strategies for future pandemics.

## Conclusion

Around one fourth of the studied participant have PTSD, even after waning of the COVID-19 pandemic. Social factors such as decrease in income and food scarcity influenced mental health issues during a pandemic. Hence, it is imperative to consider social factors such as income and food stability when addressing mental health issues during a pandemic.

## Data Availability

The original contributions presented in the study are included in the article/Supplementary material, further inquiries can be directed to the corresponding authors.

## References

[1] Hossain MM Tasnim S Sultana A Faizah F Mazumder H Zou L. Epidemiology of mental health problems in COVID-19: a review. F1000Res. (2020) 9:636. doi: 10.12688/f1000research.24457.1, PMID: 33093946 PMC7549174

[2] Chen J Zhang SX Yin A Yanez JA. Mental health symptoms during the COVID-19 pandemic in developing countries: a systematic review and meta-analysis. J Glob Health. (2022) 12:05011. doi: 10.7189/jogh.12.05011, PMID: 35604881 PMC9126304

[3] World Health Organisation. (2022) COVID-19 pandemic triggers 25% increase in prevalence of anxiety and depression worldwide. Available online at https://www.who.int/news/item/02-03-2022-covid-19-pandemic-triggers-25-increase-in-prevalence-of-anxiety-and-depression-worldwide (Accessed on 26 Feb 2025).

[4] Xiong J Lipsitz O Nasri F Lui LMW Gill H Phan L. Impact of COVID-19 pandemic on mental health in the general population: a systematic review. J Affect Disord. (2020) 277:55–64. doi: 10.1016/j.jad.2020.08.001, PMID: 32799105 PMC7413844

[5] National Crime Records Bureau. (2020) Ministry of home affairs Gov of India. Accidental deaths & suicides in India. Available online at: http://ncrb.gov.in/ (Accessed on 25 Feb 2025).

[6] Agarwala P Bhargava A Gahwai DK Negi SS Shukla P Dayama S. Epidemiological characteristics of the COVID-19 pandemic during the first and second waves in Chhattisgarh, Central India: a comparative analysis. Cureus. (2022) 14:e24131. doi: 10.7759/cureus.24131, PMID: 35573570 PMC9106595

[7] Löwe B Decker O Müller S Brähler E Schellberg D Herzog W. Validation and standardization of the generalized anxiety disorder screener (GAD-7) in the general population. Med Care. (2008) 46:266–74. doi: 10.1097/MLR.0b013e318160d093, PMID: 18388841

[8] Kroenke K Spitzer RL Williams JB. The PHQ-9. Validity of a brief depression severity measure. J Gen Intern Med. (2001) 16:606–13. doi: 10.1046/j.1525-1497.2001.016009606.x, PMID: 11556941 PMC1495268

[9] Horowitz M Wilner N Alvarez W. Impact of event scale: a measure of subjective stress. Psychosom Med. (1979) 41:209–18. doi: 10.1097/00006842-197905000-00004, PMID: 472086

[10] Sharma SK Joseph J Varkey BP Dhandapani M Varghese A Sharma S. Prevalence of anxiety and depressive symptoms during COVID-19 pandemic among the general population in India: a systematic review and meta-analysis. J Neurosci Rural Pract. (2022) 13:608–17. doi: 10.25259/JNRP-2022-1-21-R3-(2324) 36743765 PMC9894328

[11] Rehman T Singh T Sharma S Kumar J Govindan D Singh SM. Prevalence of depression and anxiety during the COVID-19 pandemic among the residents of an urban slum in North India. J Neurosci Rural Pract. (2021) 12:153–8. doi: 10.1055/s-0040-1721623, PMID: 33531775 PMC7846337

[12] Wu T Jia X Shi H Niu J Yin X Xie J. Prevalence of mental health problems during the COVID-19 pandemic: a systematic review and meta-analysis. J Affect Disord. (2021) 281:91–8. doi: 10.1016/j.jad.2020.11.117, PMID: 33310451 PMC7710473

[13] Anand V Verma L Aggarwal A Nanjundappa P Rai H. COVID-19 and psychological distress: lessons for India. PLoS One. (2021) 16:e0255683. doi: 10.1371/journal.pone.0255683, PMID: 34347847 PMC8336880

[14] Rodriguez-Rey R Garrido-Hernansaiz H Collado S. Psychological impact and associated factors during the initial stage of the coronavirus (COVID-19) pandemic among the general population in Spain. Front Psychol. (2020) 11:1540. doi: 10.3389/fpsyg.2020.01540, PMID: 32655463 PMC7325630

[15] Mazza C Ricci E Biondi S Colasanti M Ferracuti S Napoli C. A Nationwide survey of psychological distress among Italian people during the COVID-19 pandemic: immediate psychological responses and associated factors. Int J Environ Res Public Health. (2020) 17:3165. doi: 10.3390/ijerph17093165, PMID: 32370116 PMC7246819

[16] Gao J Zheng P Jia Y Chen H Mao Y Chen S. Mental health problems and social media exposure during COVID-19 outbreak. PLoS One. (2020) 15:e0231924. doi: 10.1371/journal.pone.0231924, PMID: 32298385 PMC7162477

[17] Na L Yang L Mezo PG Liu R. Age disparities in mental health during the COVID19 pandemic: the roles of resilience and coping. Soc Sci Med. (2022) 305:115031. doi: 10.1016/j.socscimed.2022.115031, PMID: 35649300 PMC9100296

[18] Marzo RR Vinay V Bahari R Chauhan S Ming DAF Nelson Fernandez SFA. Depression and anxiety in Malaysian population during third wave of the COVID-19 pandemic. Clin Epidemiol Glob Health. (2021) 12:100868. doi: 10.1016/j.cegh.2021.100868, PMID: 34549098 PMC8444444

[19] Liu N Zhang F Wei C Jia Y Shang Z Sun L. Prevalence and predictors of PTSS during COVID-19 outbreak in China hardest-hit areas: gender differences matter. Psychiatry Res. (2020) 287:112921. doi: 10.1016/j.psychres.2020.112921, PMID: 32240896 PMC7102622

[20] Cao W Fang Z Hou G Han M Xu X Dong J. The psychological impact of the COVID-19 epidemic on college students in China. Psychiatry Res. (2020) 287:112934. doi: 10.1016/j.psychres.2020.112934, PMID: 32229390 PMC7102633

[21] Wang Y Di Y Ye J Wei W. Study on the public psychological states and its related factors during the outbreak of coronavirus disease 2019 (COVID-19) in some regions of China. Psychol Health Med. (2021) 26:13–22. doi: 10.1080/13548506.2020.1746817, PMID: 32223317

[22] Joaquim RM Pinto ALCB Guatimosim RF de Paula JJ Souza Costa D Diaz AP. Bereavement and psychological distress during COVID-19 pandemics: the impact of death experience on mental health. Curr Res Behav Sci. (2021) 2:100019. doi: 10.1016/j.crbeha.2021.100019, PMID: 40115879

[23] Vindegaard N Benros ME. COVID-19 pandemic and mental health consequences: systematic review of the current evidence. Brain Behav Immun. (2020) 89:531–42. doi: 10.1016/j.bbi.2020.05.048, PMID: 32485289 PMC7260522

[24] Du J Dong L Wang T Yuan C Fu R Zhang L. Psychological symptoms among frontline healthcare workers during COVID-19 outbreak in Wuhan. Gen Hosp Psychiatry. (2020) 67:144–5. doi: 10.1016/j.genhosppsych.2020.03.011, PMID: 32381270 PMC7194721

[25] Malhotra V Javed D Bharshankar R Singh V Gautam N Mishra S. Prevalence and predictors of depression, anxiety and stress among elderly during COVID-19: a cross-sectional study from Central India. Mymensingh Med J. (2023) 32:556–66. PMID: 37002771

[26] Sundermeir SM Wolfson JA Bertoldo J Gibson DG Agarwal S Labrique AB. Food insecurity is adversely associated with psychological distress, anxiety and depression during the COVID-19 pandemic. Prev Med Rep. (2021) 24:101547. doi: 10.1016/j.pmedr.2021.101547, PMID: 34518794 PMC8425295

[27] Wolfson JA Garcia T Leung CW. Food insecurity is associated with depression, anxiety, and stress: evidence from the early days of the COVID-19 pandemic in the United States. Health Equity. (2021) 5:64–71. doi: 10.1089/heq.2020.0059, PMID: 33681691 PMC7929913

[28] Fang D Thomsen MR Nayga RM. The association between food insecurity and mental health during the COVID-19 pandemic. BMC Public Health. (2021) 21:607. doi: 10.1186/s12889-021-10631-0, PMID: 33781232 PMC8006138

[29] Allen J Smith-Carrier T Smye V Gewurtz R Isard R Goldszmidt R. Experiences of mental health and poverty in high-income countries during COVID-19: a systematic review and meta-aggregation. PLOS Ment Health. (2024) 1:e0000059. doi: 10.1371/journal.pmen.0000059 PMC12798167 41661764

[30] Adise S West AE Rezvan PH Marshall AT Betts S Kan E. Socioeconomic disadvantage and youth mental health during the COVID-19 pandemic lockdown. JAMA Netw Open. (2024) 7:e2420466. doi: 10.1001/jamanetworkopen.2024.20466, PMID: 38967921 PMC11227076

[31] Bourmistrova NW Solomon T Braude P Strawbridge R Carter B. Long-term effects of COVID-19 on mental health: a systematic review. J Affect Disord. (2022) 299:118–25. doi: 10.1016/j.jad.2021.11.031, PMID: 34798148 PMC8758130

[32] Chadda RK Deb KS. Indian family systems, collectivistic society and psychotherapy. Indian J Psychiatry. (2013) 55:S299–309. doi: 10.4103/0019-5545.105555 23858272 PMC3705700

[33] Sagar R Singh S. National Tele-Mental Health Program in India: a step towards mental health care for all? Indian J Psychiatry. (2022) 64:117–9. doi: 10.4103/indianjpsychiatry.indianjpsychiatry_145_22, PMID: 35494321 PMC9045352

[34] Rao E Patel D Saxena N Saha KB Kumar R. Ameliorating mental health issues in sickle cell disease patients: a viewpoint. Hemoglobin. (2024) 48:212–3. doi: 10.1080/03630269.2024.2356607 39329378

