# Supplementary material for: Food scarcity and decrease in income are associated with depression after COVID-19 pandemic in rural settings
Source: Front Public Health. 2025 Apr 2;13:1526300. doi: 10.3389/fpubh.2025.1526300 (PMC12002032; doi:10.3389/fpubh.2025.1526300)
Supplement: Supplementary file 1 [file Table_1.DOCX]

**Supplementary Table 1: Anxiety, Depression and Post-traumatic Stress among studied participants**

| Scale | Severity | **Number (%)** |
| --- | --- | --- |
| GAD-7 | ˃4 | 64 (14.8) |
| PHQ-9 | ˃4 | 51 (11.8) |
| IES-R | ˃23 | 113 (26.2) |
| GAD-7 | None (Score: 0-4) | 367 (85.2) |
|  | Mild (Score: 5-9) | 53 (12.3) |
|  | Moderate (Score: 10-14) | 7 (1.6) |
|  | Severe (Score >15) | 4 (0.9) |
| PHQ-9 | None (Score 0-4) | 380 (88.2) |
|  | Mild (5-9) | 45 (10.4) |
|  | Moderate (10-14) | 5 (1.2) |
|  | Moderately Severe (15-19) | 1 (0.2) |
| IES-R | None (Score: 0-23) | 318 (73.8) |
|  | Mild (Score: 24-32) | 69 (16.0) |
|  | Moderate (Score: 33-36) | 13 (3.0) |
|  | Severe (Score: >36) | 31 (7.2) |
